# Enteric or Typhoid Fever in the Very Young and Very Old

**Published:** 1892-10

**Authors:** W. B. West

**Affiliations:** Fort Worth, Texas


For Daniel's Texas Medical Journal:

### ENTERIC OR TYPHOID FEVER IN THE VERY YOUNG AND VERY OLD.

BY W. B. WEST, M. D., FORT WORTH, TEXAS.

IT HAVING been my good fortune within the past fourteen months to see two such cases, and, as there has been so much written on typhoid fever as found in Texas, I feel that it may prove of interest to some of the profession to report these two cases. However, before entering into the details, I will say, that from the writings of many of our brethren one would be led to infer that typhoid fever in Texas is not an established fact. To any of those who doubt its existence, I would say, could they have witnessed an autopsy held in Fort Worth last week, on a patient who died of typhoid fever, there would be no doubt left in their minds as to the true nature of the disease, which we treat and call typhoid fever, in Fort Worth. The examination revealed ulcers of Peyer patches,—lower portion ileum,—with ulcerations also of glands of upper portion of colon, and enlargement of all of the mesenteric glands. Typhoid fever, as is well known, is an acute infectious disease, due to a specific cause characterized by gastro-intestinal catarrh, febrile movement of continued type, varying in duration from 10 to 40 days, marked nervous symptoms, with scanty eruption of isolated, slightly elevated, rose-colored spots, disappearing upon pressure, and developed in successive crops. In infancy and childhood it does not conform closely to this type of the affection in adult life. It has only been during the first half of the present century that typhoid fever, the great fever of the present epoch, was distinctly separated from all other forms of fever, and our knowledge of its pathology placed upon a sure basis of fact.

The opinion, prior to 1840, was universally held, that infancy and childhood enjoyed immunity from it. Rilliet and Taupin, who published about the same time, independent descriptions of typhoid fever, as it appeared in children, have shown this to be erroneous; that the great number of cases of fever among children, previously described as "infantile remittent," were instances of enteric fever. Aitken, in speaking of typhoid fever in children, says: "It has now been already established that typhoid fever is by no means an infrequent disease among children, and has been often described as infantile remittent fever." (It occurs sometimes epidemically. This has been noticed at the children's hospital at Paris, and Dr. Rilliet saw an epidemic of typhoid fever in a small village near Geneva, Switzerland, which attacked children only.) Its occurence is rare during the first years of life, nevertheless it is on record at the following very early ages, namely, between two and three months, three months, six months, seven months, ten and thirteen months. (Wunderlich, Huming, Frederick, Rilliet.)

The author of a very interesting review on the typhoid fever of children, in the *British and Foreign Medico-Chirurgical Review*, for July, 1858, p. 161, mentions in his own experience the occurrence of typhoid fever in a girl one year and seven months old, and also in a boy two years of age. Murchison showed, in 1864, at the London Pathological Society, the intestines of an infant six months old, who had been attacked at the same time with her mother. The explanation of the fact, that the proportion of cases occurring in infancy is smaller than that of childhood and adolescence, is to be sought in the increased exposure to the infecting principle at the latter period. Again, enteric fever is not very common in very old persons, though there are well authenticated cases reported where the persons were sixty, seventy, eighty and ninety years old, while in this paper I will report a case in which the person was 101 years of age. The infrequency of the attack in later life, is due to the fact that such persons have probably already passed through the disease and are, therefore, partially exempt. I do not deem it necessary to go further into the history of this disease, my object being simply to report two cases, one of a very young child, and the other of a very old woman. I shall first give symptoms of child, and if there be any doubting brother, who in the face of so much authority, doubts my diagnosis, let him make his own.

Three weeks since I was called to see H—— D——, aged 10½ months. When I reached the house, the child showed every symptom of having had a chill a short while before; in fact, the chill had not fully passed off. The finger nails were blue, skin very purple, face pinched, and anxious expression, and child would nestle close to its mother, as if to keep warm. I took his temperature, found it a shade sub-normal. In about two hours I returned, when I found the temperature 101° in the axilla. On the following morning, at 8 o'clock, temperature was 99½°, and the little fellow seemed to be suffering greatly from pain in bowels, and nausea. At 6:30 p. m., temperature 102°, and patient very restless. Had slept but little, and often disturbed by cries and jactitations. Third day, at 8:15 a. m., temperature 100°, at 8 p. m. 101°, skin hot and dry, tongue much coated, with red tip and edges. The lips were parched and dry, and soon became cracked and sore. Appetite by this time was lost, would take nothing in the way of nourishment, cried for water or ice every few minutes. Diarrhœa was present and lasted until after the 15th day, when the actions became about normal. Abdomen was slightly swollen, but there was very little tenderness shown at any time upon pressure. Cough was present after the first day, and lasted throughout the attack. On the 8th day the eruption was noticed. I never saw more typical, rose-colored spots in any case I have ever treated, since leaving my native State, Virginia. The eruption extended over the abdomen, chest and back. By the 15th day the child had been reduced from a strong, healthy little fellow, to a mere shadow. During all this time the temperature was down to 99½° to 101° in the morning, with an average rise in the evening of 103°. When the temperature began to subside, it went slowly down regularly each day from a quarter to one degree; until 99½° was reached; at this point it remained for four days. On the morning of the 25th day, I found the little fellow for the first time, since the fever began, free of fever. My treatment in this case was quinine at night and sulphurous acid during the day. His digestion was at one time during the attack very much impaired. I used pepsin, bismuth, papoid, etc. The papoid acted like a charm in this case in 2 gr. doses every three hours. The little fellow at this writing, like Richard of old, is himself again.

The old lady, Mrs.———, aged 101 years, was taken with typhoid fever in June, 1891. Up to the time she was attacked she was in splendid health for one of her *tender* years. There was nothing in this case out of the ordinary, except her age, which, I think, is the oldest on record. The attack lasted twenty-one days. Severe frontal headache, with slight pains in back of the head, and marked pains in small of the back, lasting for nearly two weeks. Constipation was present at first, followed by diarrhœa. Rose-colored spots were present, but not in great numbers. In fact, she had all of the symptoms of typhoid fever. I found great difficulty in administering medicines and nourishment, due to her childishness, her mind having been very much impaired by age. She was convinced from the first that she had typhoid fever, and said she knew she could not recover, and did not wish to take any medicines or nourishment. I was compelled, after using every other means, to force her to take them. The Sisters of Charity, who had her in charge, would have to force open her mouth and pour the nourishment down, as if she were a spoiled child. I found that very small doses were sufficient in her case. She made a good and rapid recovery, and is at this writing living and enjoying fine health, at the good old age of 102 years. When last I saw her, she assured me that she was cutting two new teeth; however, I am not prepared to vouch for the latter.

